# Assessment of Amyloid Deposition in Patients With Probable REM Sleep Behavior Disorder as a Prodromal Symptom of Dementia With Lewy Bodies Using PiB-PET

**DOI:** 10.3389/fneur.2019.00671

**Published:** 2019-06-25

**Authors:** Ryota Kobayashi, Hiroshi Hayashi, Shinobu Kawakatsu, Nobuyuki Okamura, Masanori Yoshioka, Koichi Otani

**Affiliations:** ^1^Department of Psychiatry, Yamagata University School of Medicine, Yamagata, Japan; ^2^Department of Neuropsychiatry, Aizu Medical Center, Fukushima Medical University, Aizuwakamatsu, Japan; ^3^Department of Pharmacology, Faculty of Medicine, Tohoku Medical and Pharmaceutical University, Sendai, Japan; ^4^Department of Radiology, Yamagata University Hospital, Yamagata, Japan

**Keywords:** Dementia with Lewy bodies, REM sleep behavior disorder, Alzheimer's disease, amyloid-β, prodromal stage, PiB-PET

## Abstract

**Introduction:** Dementia with Lewy bodies (DLB) often exhibits REM sleep behavior disorder (RBD) at its prodromal stage. Meanwhile, DLB is often comorbid with Alzheimer's disease (AD)-type pathology. In typical AD, amyloid-β deposition begins considerably before the onset of dementia and has already reached a plateau at the stage of mild cognitive impairment. However, it is not known when amyloid accumulation starts in DLB with AD-type pathology. In the present study, we examined amyloid deposition in patients with RBD as a prodromal symptom of DLB using [11C]-Pittsburgh compound B positron emission tomography (PiB-PET).

**Methods:** The subjects were 12 patients with probable RBD as diagnosed by the Japanese RBD screening questionnaire. They also showed abnormality in 123I-metaiodobenzylguanidine myocardial scintigraphy, a biomarker for DLB. For comparison, 11 patients with probable DLB were included. Applying PMOD software to the PiB-PET images, the global cortical distribution volume ratio was calculated and a ratio >1.3 was regarded as PiB-positive.

**Results:** Two of the RBD patients (16.7%) and eight of the DLB patients (72.7%) were PiB-positive. The amyloid-positive rate was significantly lower in the RBD group than in the DLB group (*P* = 0.012).

**Conclusion:** The prevalence of amyloid deposition in RBD as a prodromal symptom of DLB was significantly lower than that in DLB, suggesting that amyloid accumulation does not always begin at the early stage of DLB.

## Introduction

Dementia with Lewy bodies (DLB) is the second most common dementia after Alzheimer's disease (AD) (1). DLB reduces quality of life and lifespan to a greater extent than AD (2); therefore, early diagnosis of and intervention for DLB are important. Recently, prodromal symptoms of DLB have attracted attention, and REM sleep behavior disorder (RBD), dysautonomia, olfactory dysfunction, and psychiatric symptoms have been reported to precede the onset of DLB by several years (3). RBD is a parasomnia characterized by dream-enacting behaviors without muscle atonia during REM sleep (4). Idiopathic patients with RBD have a high affinity for DLB as reflected by abnormality in 123I-metaiodobenzylguanidine (MIBG) myocardial scintigraphy (5), a biomarker for DLB (1), and have a high possibility of developing Lewy body disease (LBD), which includes DLB. In observational studies, the median interval between the diagnosis of RBD and that of a defined neurodegenerative syndrome was 4 years (6).

Patients with DLB are often accompanied by AD-type pathology [e.g., (7, 8)], and this is comorbidity is associated with a worse prognosis [e.g., (8, 9)]. Therefore, early detection of and intervention for AD-type pathology may be beneficial (9). Since [11C]-Pittsburgh compound B positron emission tomography (PiB-PET) has emerged as a standard amyloid-β (Aβ) imaging method, many studies have demonstrated amyloid accumulation in DLB. According to a systematic review of PiB-PET studies, the positive rate of Aβ accumulation in patients with DLB is as high as 68% (10). This finding suggests a high rate of comorbid AD-type pathology in DLB.

In typical AD, deposition of Aβ starts ~15 years prior to the onset of symptoms, and amyloid PET shows positive findings in the mild cognitive impairment (MCI) phase or even in the preclinical phase of the disease (11). Furthermore, Aβ deposition has reached almost a plateau at the stage of MCI (11). On the other hand, the onset of Aβ accumulation in the course of DLB is not known. However, in light of the high rate of amyloid positivity in DLB mentioned above (10), it is not surprising that Aβ accumulation has already started in the prodromal stage of DLB.

The purpose of this study was to investigate amyloid deposition in RBD as a prodromal symptom of DLB using PiB-PET. We performed PiB-PET in patients with probable RBD as a prodromal symptom of DLB (MIBG myocardial scintigraphy confirmed), and in those with probable DLB (MIBG myocardial scintigraphy and/or dopamine transporter imaging confirmed), and compared the rates of amyloid positivity between these two groups.

## Methods

### Subjects

The subjects were 12 patients with probable RBD who visited the Department of Psychiatry at Yamagata University Hospital between February 2014 and March 2019. The diagnosis of probable RBD was made using the Japanese RBD screening questionnaire (RBDSQ-J) (4), which has high sensitivity and specificity for idiopathic RBD. Furthermore, all patients showed abnormal findings in MIBG myocardial scintigraphy according to the criteria of Nakajima et al. (12). They also received general physical, psychiatric and neurological examinations, extensive laboratory tests, and magnetic resonance imaging. Patients with a history of cerebrovascular diseases, neurodegenerative diseases, diabetes mellitus, and psychiatric diseases were excluded. Absence of visual hallucination, cognitive fluctuation or Parkinson's symptoms was confirmed by detailed clinical examinations. For comparison, 11 patients with probable DLB from our hospital were included. These patients showed abnormal findings in MIBG myocardial scintigraphy and/ or dopamine transporter imaging, and fulfilled the criteria for probable DLB developed by McKeith et al. (1). The Ethics Committee of Yamagata University School of Medicine approved the present study, and written informed consent for participation was obtained from all patients.

### PET Imaging

[11C]-PiB was synthesized at our institution's PET facility as described previously (11). PET scans were performed using a PET/Computed Tomography scanner (Biograph mCT, Siemens Healthineers, Tokyo, Japan) in three-dimensional scanning mode. [11C]-PiB was injected into an antecubital vein at a mean (SD) dose of 555 (185) MBq (10 MBq/kg body weight), followed immediately by a 70-min dynamic acquisition. The PET images were reconstructed into 25 time frames (6 × 10, 3 × 20, 2 × 60, 2 × 180, and 12 × 300 s) using the standard ordered subset expectation maximization algorithm (subset 21, iteration 4), point spread function and time of flight. These images were analyzed with PMOD software (version 3.409, PMOD Technologies Ltd., Zurich, Switzerland). Global cortical PiB retention was calculated using the Logan graphical analysis method, with the cerebellar cortex as the reference tissue input function, and expressed as the distribution volume ratio (DVR) (13). A global cortical DVR >1.3 was regarded as PiB-positive (13).

### Apolipoprotein E (APOE) Genotyping

APOE genotypes were determined using restriction fragment length polymorphism analysis. One patient from each group refused a blood sampling for genotyping.

### Statistical Analysis

Differences in demographic and clinical data of the subjects were tested using Student's *t*-test or Fisher's exact test, as appropriate. Fisher's exact test was used to compare the PiB-positive rate between the RBD group and the DLB group. Statistical analyses were performed using SPSS software (version 25, IBM, New York, USA) and a *P*-value <0.05 was considered statistically significant.

## Results

Demographic and clinical data of the subjects are shown in Table 1. There was no significant difference in age, duration of education or presence of the APOE ε4-allele between the RBD and DLB groups. Scores of the Mini Mental State Examination were significantly (*P* < 0.001) higher in the RBD group than in the DLB group. There was no significant difference in the frequency of the APOE ε4-allele, a known risk factor for AD pathology (9).

**Table 1:** Demographic and clinical data of subjects.

	**RBD (*n* = 12)**	**DLB (*n* = 11)**	***P***-**value**
Age (years)	74.0 (5.2)	77.0 (5.3)	0.19
Sex (male/female)	10/ 2	4/ 7	0.036
Education (years)	12.8 (1.6)	12.1 (1.3)	0.236
Duration (years)	6.3 (3.3)	2.0 (1.2)	
MMSE (/30)	28.6 (1.5)	21.5 (3.7)	0.001
APOE ε4-allele present	2/11 (18%)	4/10 (40%)	0.361

*Values are presented as mean (SD) or number (%)*.

*Differences between groups were assessed using Student's t-test (age, education, and MMSE) and Fisher's exact test (sex and APOE ε4-allele)*.

*APOE, apolipoprotein E; DLB, dementia with Lewy bodies; MMSE, Mini Mental State Examination; RBD, REM sleep behavior disorder*.

**Figure 1:**
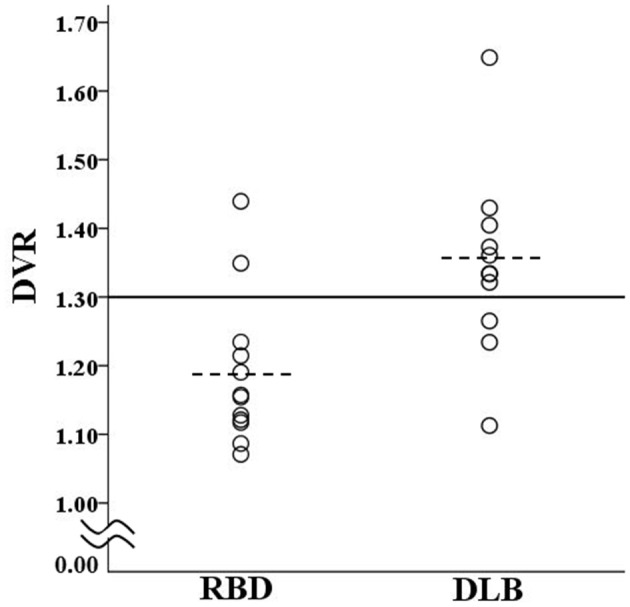
Scatter plot of DVR for patients in the RBD group and DLB group. The solid line shows the cut off value (1.3) for discrimination between PiB-positive and PiB-negative. The dotted lines show the mean values of the RBD group (1.19) and DLB group (1.35). DLB, dementia with Lewy bodies; DVR, global cortical distribution volume ratio; PiB, Pittsburgh compound B; RBD, REM sleep behavior disorder.

Based on the global cortical DVR, two of the RBD patients (16.7%) and eight of the DLB patients (72.7%) were classified as PiB-positive (Figure 1). The prevalence of amyloid deposition was significantly (*P* = 0.012) lower in the RBD group than in the DLB group. The detailed demographic, cognitive, genetic, and PiB-PET data of individual subjects are provided in Table S1.

## Discussion

In the present study, the amyloid-positive rate was 16.7% in RBD patients, while it was 72.7% in DLB patients. The result for DLB is in line with a previous systematic review reporting a 68% amyloid-positive rate in DLB (10).

In light of the high rate of concomitant AD-type pathology in DLB, it was expected that amyloid deposition in DLB is already observed in a considerable degree before onset of the disease. If this was the case, the amyloid-positive rate of the RBD group would have been similar to that of the DLB group. However, the amyloid-positive rate was unexpectedly low in the RBD group. Similar results were shown in an analysis of Alzheimer's biomarkers in cerebrospinal fluid, i.e., Aβ42 values were significantly higher in prodromal DLB patients than in those with DLB patients (14), indicating an apparently lower degree of amyloid accumulation in the former. In relation to this, according to a systematic review of amyloid PET in LBDs other than DLB, the amyloid-positive rate was 34% in the patient group of Parkinson's disease (PD) with dementia, while it was only 5% in that of PD with MCI (10). Namely, in the PD spectrum amyloid deposits are not likely to reach a plateau at the MCI stage. These findings along with our result suggest that amyloid deposition pathology in LBD, including DLB, does not start as early as in AD.

In the treatment of DLB, not only Lewy pathology but also the coexistence of complex AD-type pathology causes serious problems (9). Therefore, to clarify the time course of AD-type pathology in DLB is extremely important from a treatment perspective. The present study suggesting low amyloid deposition at the prodromal stage of DLB should be followed by studies attempting to elucidate the starting point of amyloid accumulation in the course of DLB. Those studies may provide valuable information on the time to begin the treatment for amyloid pathology in DLB.

There are several limitations in the present study. First, although the patients of the RBD group were screened by the RBDSQ-J with high sensitivity and specificity, they did not receive polysomnography and, therefore, remained “patients with probable RBD.” Second, the patients of the RBD group might be in the prodromal stage of α-synucleinopathies other than DLB. However, a previous report showed that RBD patients have the highest risk of developing DLB among neurodegenerative disorders during follow-up (5), suggesting that our RBD patients include a high proportion of patients who later develop DLB. In either case, it is necessary to conduct a follow-up examination of our RBD patients to confirm phenoconversion to DLB. Third, males were over-presented in the RBD group, which was inevitable because of the known sex difference in the frequency of this disorder (4, 6). As far as we know, there have been no data suggesting sex differences in the rate and degree of amyloid positivity in DLB, the possibility that the skewed sex distribution was involved in the present result cannot be excluded entirely. Fourth, we suggested that amyloid accumulation does not occur in the RBD group based on the amyloid-positive rate of 16.7%, but to be exact it should be confirmed that this rate is comparable to that in a healthy control group, which was not included in this study. Fifth, the confirmation of the absence of dementia in the RBD group was based exclusively on the MMSE. Finally, the present study was a pilot study with a relatively few number of subjects, necessitating a replication study with a larger number of subjects.

In conclusion, the prevalence of amyloid deposition in RBD as a prodromal symptom of DLB was significantly lower than that in DLB. This result suggests that amyloid accumulation does not always precede the onset of cognitive decline in patients with DLB.

## Data Availability

All datasets generated for this study are included in the manuscript and/or the Supplementary Files.

## Ethics Statement

This study was carried out in accordance with the recommendations of International Committee of Medical Journal Editors with written informed consent from all subjects. All subjects gave written informed consent in accordance with the Declaration of Helsinki. The protocol was approved by the Ethics Committee of Yamagata University School of Medicine.

## Author Contributions

RK conceptualized the study, conducted neuropsychological examinations, analyzed the data, and drafted the manuscript. HH and SK conducted neuropsychological examinations and revised the manuscript. NO analyzed the data and drafted the manuscript. MY conducted neuroradiological examinations and was involved in drafting the manuscript. KO encouraged the study and revised the manuscript. All authors have read and approved the final version of this manuscript.

### Conflict of Interest Statement

The authors declare that the research was conducted in the absence of any commercial or financial relationships that could be construed as a potential conflict of interest.
