# Supplementary material for: Assessment of Amyloid Deposition in Patients With Probable REM Sleep Behavior Disorder as a Prodromal Symptom of Dementia With Lewy Bodies Using PiB-PET
Source: Front Neurol. 2019 Jun 25;10:671. doi: 10.3389/fneur.2019.00671 (PMC6603167; doi:10.3389/fneur.2019.00671)
Supplement: Supplementary file 1 [file Table_1.docx]

| Table S1 The detailed demographic, cognitive, genetic and PiB-PET data of individual subjects | | | | | | |
| --- | --- | --- | --- | --- | --- | --- |
|  | Case No. | Age  (years) | Sex | Mini Mental State Examination (/30) | Apolipoprotein E genotype | Global cortical  distribution volume ratio |
| REM sleep behavior disorder | 1 | 73.8 | Male | 28 | 3/3 | 1.12 |
|  | 2 | 75.3 | Female | 30 | Not available | 1.19 |
|  | 3 | 76.6 | Male | 26 | 3/3 | 1.15 |
|  | 4 | 78.3 | Male | 26 | 3/4 | 1.23 |
|  | 5 | 69.8 | Female | 30 | 3/4 | 1.12 |
|  | 6 | 81.2 | Male | 30 | 3/3 | 1.44 |
|  | 7 | 75.8 | Male | 27 | 3/3 | 1.21 |
|  | 8 | 78.9 | Male | 28 | 3/3 | 1.35 |
|  | 9 | 75.4 | Male | 29 | 3/3 | 1.09 |
|  | 10 | 73.7 | Male | 30 | 3/3 | 1.16 |
|  | 11 | 62.9 | Male | 30 | 3/3 | 1.07 |
|  | 12 | 66.7 | Male | 29 | 3/3 | 1.13 |
| Dementia with Lewy bodies | 13 | 72.4 | Female | 25 | 3/3 | 1.36 |
|  | 14 | 74.8 | Female | 22 | 3/4 | 1.33 |
|  | 15 | 75.3 | Female | 14 | 3/3 | 1.32 |
|  | 16 | 82.5 | Female | 20 | 3/3 | 1.42 |
|  | 17 | 77.8 | Male | 26 | 3/3 | 1.33 |
|  | 18 | 84.7 | Female | 24 | Not available | 1.23 |
|  | 19 | 85.4 | Female | 22 | 3/4 | 1.37 |
|  | 20 | 69.6 | Male | 25 | 3/3 | 1.40 |
|  | 21 | 76.4 | Male | 19 | 3/4 | 1.65 |
|  | 22 | 71.3 | Male | 17 | 3/3 | 1.11 |
|  | 23 | 77.3 | Female | 23 | 3/4 | 1.27 |
